# *Emmonsia helica* Infection in HIV-Infected Man, California, USA

**DOI:** 10.3201/eid2401.170558

**Published:** 2018-01

**Authors:** Martin Rofael, Ilan S. Schwartz, Lynne Sigler, Li K. Kong, Nicholas Nelson

**Affiliations:** Highland Hospital, Oakland, California, USA (M. Rofael, L.K. Kong, N. Nelson);; University of Manitoba, Winnipeg, Manitoba, Canada (I.S. Schwartz);; University of Texas Health San Antonio, San Antonio, Texas, USA (I.S. Schwartz);; University of Alberta, Edmonton, Alberta, Canada (L. Sigler)

**Keywords:** emmonsiosis, AIDS, mycosis, opportunistic infection, endemic dimorphic fungi, viruses, HIV, California

## Abstract

*Emmonsia*-like fungi have rarely been reported from North America. We report a fatal case of *E. helica* infection in a man with advanced HIV infection from California, USA, who had progressive respiratory failure and a brain abscess.

In January 2016, a 40-year-old man sought care at a hospital in Alameda County, California, USA, with a 2-week history of progressive cough, dyspnea, pleuritic chest pain, and headache associated with fevers, chills, and night sweats. He had lost 45 kg during the past month. He had a history of inconsistently treated HIV infection and a 10-pack-per-year smoking history. He had emigrated from Mexico 10 years earlier, lived in the East Bay, and had traveled to the Central Valley of California.

On examination, he was cachectic, afebrile, hypoxic, and tachypneic. He had oral thrush, bilateral lower lobe crackles, and decreased breath sounds. The rest of the examination, including neurologic and skin assessments, was unremarkable.

Chest radiograph and computerized tomographic scan showed diffuse micronodularities with cavitary lesions in both lungs (Figure, panels A, B). Magnetic resonance imaging of the brain revealed a 6-mm ring-enhancing cerebellar lesion (Figure, panel C).

**Figure:**
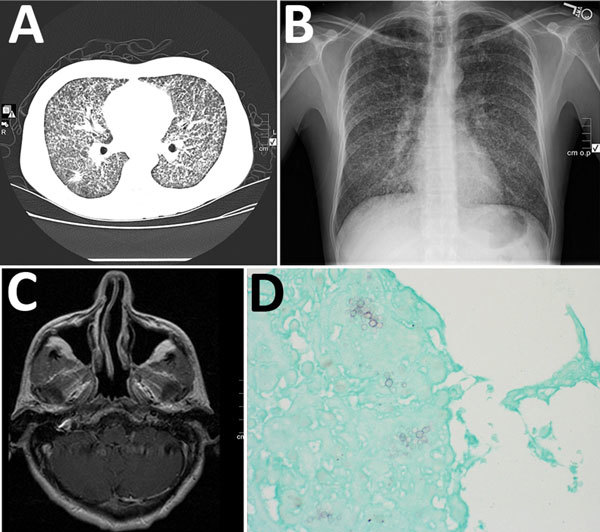
*Emmonsia helica* infection in an immunocompromised man, California, USA, 2016. A) Chest radiograph with diffuse micronodularities throughout both lung fields. B) Computed tomographic scan with diffuse micronodular pulmonary disease. C) Axial magnetic resonance image with 6-mm ring-enhancing lesion in the right cerebellum adjacent to the fourth ventricle. D) Grocott’s methenamine-silver stain showing broad-based budding yeast. Original magnification ×400.

Laboratory results revealed a CD4 count and viral load of 5 cells/μL and 15,000 copies/mL, respectively. Blood leukocyte count was 14.9 × 10^9^ cells/μL (reference 4.5–11 × 10^9^ cells/μL), and hemoglobin was 11 g/dL (reference 13.5–17.5 g/dL). Lactate dehydrogenase, alkaline phosphatase, and γ-glutamyl transferase were 284 units/L (reference 125–243 U/L), 148 units/L (reference 38–126 U/L), and 242 units/L (reference 3–95 U/L), respectively. Cerebrospinal fluid analyses, including cell count, protein, and glucose measurements, showed standard results.

Molecular testing of bronchoalveolar lavage (BAL) fluid for respiratory viruses showed negative results. Serologic test results for blood and urine for coccidioidomycosis, cryptococcosis, toxoplasmosis, and tuberculosis were negative, but the *Histoplasma* galactomannan urine antigen test (MiraVista, Indianapolis, IN, USA) result was positive at >25 ng/mL (reference <0.5 ng/mL). Examination of cerebrospinal fluid with Gram, acid-fast, and India ink stains and aerobic, mycobacterial, and fungal cultures were negative for organisms. Cytopathologic examination of BAL fluid and lung biopsy samples showed nonnecrotizing granulomas with hematoxylin and eosin stain and both hyphal and yeast forms with fungal stains. The yeasts exhibited multiple budding with broad bases (Figure, panel D). No organisms were seen with an acid-fast stain. Multiple sputum cultures were negative for mycobacteria.

Empiric treatment was initiated for coccidioidomycosis, *Pneumocystis jirovecii* pneumonia, tuberculosis, and bacterial sepsis with intravenous fluconazole; trimethoprim–sulfamethoxazole with steroids; rifampin, isoniazid, pyrazinamide, and ethambutol; and broad-spectrum antimicrobial drugs. On hospital day 2, the patient had hypoxic respiratory arrest and was intubated. Antifungal drugs were changed to micafungin on day 3 and then to liposomal amphotericin B (5 mg/kg/day) on day 6, and he was extubated later that day. Tuberculosis therapy was discontinued on day 15. Antiretroviral therapy was held because of concern that immune reconstitution might worsen the patient’s cerebellar lesion. A spontaneous pneumothorax and respiratory failure developed that required reintubation on day 31. His family chose comfort care, and antifungal therapy was stopped on day 41. He died on day 43; autopsy was declined.

We sent a mold grown from BAL to a mycology reference laboratory for identification. On microscopic examination of the mold phase, conidia were absent in all subcultures. DNA sequences of the D1/D2 region of the large subunit and internal transcribed spacer region of the ribosomal RNA gene were compared with GenBank nBLAST database (https://blast.ncbi.nlm.nih.gov/Blast.cgi?PAGE_TYPE=BlastSearch); sequence similarities of 100% and 99%, respectively, were demonstrated for *Emmonsia helica* strains (including UAMH 3398, UAMH 10539, and UAMH 10593; UAMH Centre for Global Microfungal Biodiversity, University of Toronto, Toronto, Ontario, Canada).

We conducted antifungal susceptibility testing of the mold phase. MICs (μg/mL) to fluconazole, itraconazole, posaconazole, voriconazole, and amphotericin B were 8, 0.125, 0.125, <0.03, and 0.06, respectively.

*Emmonsia*-like fungi are an emerging group of pathogens reported globally, which predominantly cause disseminated disease of immunocompromised persons (*1*). One of these, *E. helica*, was originally recored from North America (*2*). The first reported case occurred in Alberta, Canada, in 1970 in a farmer with a fatal pneumonia and encephalitis syndrome (*3*). A fungal pathogen isolated postmortem from brain and lung tissue was initially identified as *Blastomyces dermatitidis* on the basis of serologic and histopathologic findings, but its features in culture were atypical (*3*). In 2015, Sigler determined that this isolate belonged to a new *Emmonsia*-like species, which she described as *E. helica* (*3*). Another fatal case of *Emmonsia* infection was reported from California in a patient after an orthotopic liver transplant (*4*). An isolate from that patient also was confirmed as *E. helica* (I. Schwartz et al., unpub. data).

Although the travel history for the second case-patient was not reported (*4*) and the patient in this report had resided in Mexico, these cases suggest that the area of endemicity of *E. helica* may include California. This finding is further supported by 2 other fatal cases of atypical mycoses reported in HIV-infected men from California (*5*); histopathologic findings of hyphae and multiple budding yeasts were consistent with *E. helica* (I. Schwartz et al., unpub. data). Investigations are under way to characterize the geographic and host range of *E. helica* and to clarify the phylogenetic relationships among members of the family *Ajellomycetaceae* comprising the genera *Emmonsia*, *Blastomyces*, *Histoplasma* and others because recent studies have uncovered far greater complexity than previously supposed (*1*,*6*).
